# Inhibitory effects of magnolol and honokiol on human calcitonin aggregation

**DOI:** 10.1038/srep13556

**Published:** 2015-09-01

**Authors:** Caiao Guo, Liang Ma, Yudan Zhao, Anlin Peng, Biao Cheng, Qiaoqiao Zhou, Ling Zheng, Kun Huang

**Affiliations:** 1 Tongji School of Pharmacy, Huazhong University of Science and Technology, Wuhan, Hubei, P. R. China, 430030; 2 Dept. of Pharmacy, The Third Hospital of Wuhan, Wuhan, Hubei, P. R. China, 430060; 3 Dept. of Pharmacy, Central Hospital of Wuhan, Wuhan, Hubei, P. R. China, 430014; 4 College of Life Sciences, Wuhan University, Wuhan, Hubei, P. R. China, 430072; 5 Centre for Biomedicine Research, Wuhan Institute of Biotechnology, Wuhan, Hubei, P. R. China, 430075

## Abstract

Amyloid formation is associated with multiple amyloidosis diseases. Human calcitonin (hCT) is a typical amyloidogenic peptide, its aggregation is associated with medullary carcinoma of the thyroid (MTC), and also limits its clinical application. *Magnolia officinalis* is a traditional Chinese herbal medicine; its two major polyphenol components, magnolol (Mag) and honokiol (Hon), have displayed multiple functions. Polyphenols like flavonoids and their derivatives have been extensively studied as amyloid inhibitors. However, the anti-amyloidogenic property of a biphenyl backbone containing polyphenols such as Mag and Hon has not been reported. In this study, these two compounds were tested for their effects on hCT aggregation. We found that Mag and Hon both inhibited the amyloid formation of hCT, whereas Mag showed a stronger inhibitory effect; moreover, they both dose-dependently disassembled preformed hCT aggregates. Further immuno-dot blot and dynamic light scattering studies suggested Mag and Hon suppressed the aggregation of hCT both at the oligomerization and the fibrillation stages, while MTT-based and dye-leakage assays demonstrated that Mag and Hon effectively reduced cytotoxicity caused by hCT aggregates. Furthermore, isothermal titration calorimetry indicated Mag and Hon both interact with hCT. Together, our study suggested a potential anti-amyloidogenic property of these two compounds and their structure related derivatives.

Amyloidogenic proteins are capable of misfolding and assembling into amyloid deposits which are considered to be important causative factors of amyloid diseases such as Alzheimer’s disease, Parkinson’s disease and type 2 diabetes mellitus^1^,^2^,^3^. In pathological situations, amyloidogenic proteins aggregate into oligomers, followed by forming extensive linear fibrils, which is accompanied with a structural transition into β-sheet-rich structures^4^,^5^,^6^,^7^,^8^,^9^. Studies have shown that the oligomeric intermediates are the most toxic species during amyloid aggregation^10^,^11^,^12^,^13^, which induce cell apoptosis mostly by penetrating the lipid bilayer of the cell membrane^14^,^15^,^16^. Preventing amyloid proteins from aggregating into toxic conformers has thus become a strategy to prevent or treat amyloid diseases^17^.

Human calcitonin (hCT) is a 32-residue blood calcium and bone resorption regulating peptide secreted by the C cells of the thyroid (Fig. 1A)^18^. Originally, hCT was used to treat osteoporosis and Paget’s disease^19^,^20^, however, due to its high intrinsic tendency to aggregate and the low bioactivity as the result of aggregation, the clinical application of hCT has been discontinued by FDA^21^,^22^. Moreover, amyloid deposits of hCT have been discovered in patients with medullary carcinoma of the thyroid (MTC), indicating an association between MTC and hCT aggregation^23^,^24^. Therefore, salmon calcitonin (sCT), which has much lower aggregation propensity but only shares 50% homology to hCT, is clinically used instead^25^. However, hCT has a much higher potency than sCT^26^, and sCT administration can cause side effects like anorexia and vomiting^27^,^28^. Inhibiting hCT aggregation is thus of great importance both for pharmaceutical preparation of hCT *in vitro* and for the treatment of MTC.

Great efforts have been made to identify inhibitors that suppress the aggregation of amyloidogenic proteins^17^,^29^,^30^,^31^,^32^,^33^, among which, compounds derived from herbal medicines have been extensively reported^34^,^35^. *Magnolia officinalis* is a traditional Chinese herbal medicine with multiple pharmaceutical activities including eliminating damp and phlegm, relieving distension^36^, and potential anti-tumor properties^37^. Two polyphenols derived from *Magnolia officinalis*, magnolol (Mag) and honokiol (Hon), are its major effective ingredients, which are known to have anti-oxidation^37^,^38^, anti-tumor^39^,^40^,^41^, anti-inflammatory^42^,^43^ and neuroprotective^44^ properties. Recent studies also suggested Mag and Hon exhibited beneficial effects on amyloid-β induced cytotoxicity^45^,^46^.

On the other hand, polyphenols, which have multiple aromatic phenolic rings, have been regarded as a class of potential amyloid inhibitors^47^,^48^. Flavonoids along with their derivatives are the most studied polyphenols, for example, (−)-epigallocatechin 3-gallate (EGCG), a derivative of flavanone, has been extensively studied for its anti-amyloidogenic activity on α-synuclein^49^, SEVI^50^, islet amyloid polypeptide^51^ and amyloid β^52^,^53^, and is currently undergoing a phase II/III clinical study to treat Alzheimer’s disease^54^. Other polyphenols such as curcumin, caffeic acid, have also been reported^17^. However, the anti-amyloidogenic property of polyphenols with a biphenyl backbone, such as Mag and Hon (Fig. 1C,D), have not been determined. Here, hCT was used as a model to test their anti-amyloid aggregation properties.

## Results

### Magnolol and honokiol inhibited the amyloid formation of human calcitonin

Thioflavin-T (ThT) fluorescence based assay was used to monitor amyloid formation of hCT. 25 μM hCT gave a strong ThT emission, reaching the plateau stage after 40 h incubation with a lag time of 22.84 ± 2.03 h (Fig. 2A,B). As a control, addition of an equimolar amount of EGCG significantly inhibited the aggregation of hCT (Fig. 2A,B), which agrees with a previous report^55^.

Mag and Hon both inhibited hCT aggregation in a dose-dependent manner. The addition of an equimolar amount of Mag extended the lag time to 33.34 ± 1.67 h (*P* < 0.05) without significantly reducing the maximum ThT fluorescence intensity (Fig. 2A). However, in the presence of 3-fold molar excess of Mag, the maximum ThT fluorescence intensity was substantially decreased to 42% of that of the control, accompanied with a prolonged lag time of 47.19 ± 3.62 h (*P* < 0.05). When the molar ratio was further increased to 5:1, the aggregation of hCT was significantly inhibited, with a lag time of 57.44 ± 5.59 h (*P* < 0.05). Compared to Mag, Hon was less potent on inhibiting hCT aggregation. The presence of an equimolar amount of Hon slightly decreased the maximum ThT fluorescence intensity to 74% of that of the control, without prolonging the lag time (Fig. 2B). The addition of 3-fold and 5-fold amount of Hon showed stronger inhibitory effects, which decreased maximum ThT fluorescence intensity by 35% and 55%, with the lag time prolonged to 37.78 ± 1.48 h (*P* < 0.05) and 36.32 ± 4.67 h (*P* < 0.05), respectively.

Transmission electron microscopy (TEM) was then used to observe the morphology of hCT aggregates to corroborate the ThT fluorescence assay results^56^. After 72 h of incubation, hCT formed extensive linear fibrils (Fig. 2C). Addition of an equimolar amount of EGCG significantly inhibited the fibril formation of hCT (Fig. 2D), which is consistent with a previous report^55^. In the presence of Mag and Hon, hCT showed no obvious fibrils after 72 h of incubation (Fig. 2E–J). Amorphous aggregates were observed for hCT samples co-incubated with Mag and Hon at molar ratios of 1:1 or 3:1 (Fig. 2E,F,H,I), whereas fewer aggregates were observed in the presence of 5-fold molar amount of Mag or Hon (Fig. 2G,J).

### Magnolol and honokiol inhibited both oligomerization and fibrillation of hCT

The effects of Mag and Hon on hCT oligomerization and fibrillation were further determined by immuno-dot blot. A11 and OC antibodies were applied to detect the formation of oligomers and fibrils, respectively^57^,^58^. For hCT, A11-positive oligomers were observed after 24 h of incubation, while OC-positive fibrils emerged after 36 h incubation (Fig. 3). In the presence of equimolar amount of EGCG, neither A11-positive oligomers nor OC-positive fibrils were observed after 72 h of incubation, which agrees with the TEM results (Fig. 2D).

Mag and Hon both significantly suppressed the fibrillation of hCT. In the presence of two compounds, no OC-positive fibrils were observed during the incubation, except in the presence of an equimolar amount of Mag, where small amount of fibrils were detected after 72 h (Fig. 3). Mag and Hon also inhibited the oligomerization of hCT dose-dependently. In the presence of equimolar amounts of Mag and Hon, A11-positive oligomers were only detected after 48 h of incubation (Fig. 3). When the molar ratios of these two compounds were further increased to 3:1 and 5:1, essentially no oligomer was detected by the A11 antibody after 72 h (Fig. 3).

The particle size distribution of amyloid aggregates was further determined by dynamic light scattering (DLS). For hCT, the observed average diameter of aggregates was 580 nm after 24 h of incubation, and was increased to over 5000 nm after 72 h (Fig. 4). The addition of an equimolar amount of EGCG decreased the average diameter to 1300 nm after 72 h (Fig. 4). The presence of equimolar amounts of Mag and Hon showed no significant effect on the size of hCT aggregates, which displayed an average diameter of 2000 nm and 4200 nm after 72 h, respectively (Fig. 4). However, when a 5-fold molar excess of Mag and Hon was added, the average diameters of hCT aggregates were significantly decreased to 680 and 1630 nm after 72 h, respectively (Fig. 4).

### Magnolol and honokiol reduced the hCT aggregation resulted cytotoxicity and membrane disruption

The effects of compounds on hCT aggregation induced cytotoxicity were studied on SH-SY5Y cells by MTT assay. The presence of 10 μM hCT for 24 h reduced cell viability to 72% of that of untreated cells (Fig. 5A). The addition of an equimolar amount of EGCG significantly increased cell viability to 177% (*P* < 0.05). The presence of Mag and Hon both reduced the cytotoxicity to different extents (Fig. 5A). The addition of an equimolar amount of Mag increased cell viability to 88% (*P* < 0.05). When the molar ratios of Mag were increased to 3:1 and 5:1, the cell viabilities were increased to 100% and 111%, respectively (*P* < 0.05). In contrast, the presence of an equimolar amount of Hon was less effective than Mag on reducing cytotoxicity with a cell viability of 82% (*P* < 0.05). At higher concentrations (3-fold and 5-fold), Hon increased cell viabilities to 89% and 93%, respectively (*P* < 0.05).

Since all three compounds displayed proliferation effects on SH-SY5Y cells (Fig. 5A & S1), to determine whether these compounds also reduced cytotoxicity through inhibiting aggregation, we further applied fluorescence dye leakage assay to determine the effects of Mag and Hon on membrane disruption caused by hCT in a model membrane system. The addition of 1 μM hCT resulted in 26% membrane disruption compared with the vesicles treated with Triton X-100, which was set as 100% (*P* < 0.05; Fig. 5B). Addition an equimolar amount of EGCG significantly decreased dye leakage to 2% (*P* < 0.05). The presence of Mag and Hon both dose-dependently protected the membrane. Equimolar amount of two compounds slightly attenuated the membrane penetration to 14% and 18%, respectively (*P* < 0.05; Fig. 5B), while higher concentrations of compounds (3-fold and 5-fold) almost completely abolished membrane disruption (*P* < 0.05; Fig. 5B).

### Magnolol and honokiol can disaggregate preformed hCT aggregates

The efficacy of Mag and Hon to disaggregate preformed hCT aggregates was determined by ThT fluorescence assay. hCT was pre-incubated for 48 h to form aggregates, different amounts of compounds were then added. Adding equimolar amounts of Mag and Hon showed no obvious disaggregation effect, while the presence of 3-fold amount of either compound prevented further aggregation of hCT (Fig. 6A,B). When the molar ratio of Mag and Hon was further increased to 5:1, the hCT aggregates were disassembled with ThT fluorescence intensity remarkably reduced to 53% and 55% of untreated control after 48 h of incubation, respectively (Fig. 7). The morphology of hCT aggregates after disaggregation was then observed under TEM. Addition of Mag and Hon disaggregated preformed hCT fibrils into small amorphous aggregates (Fig. 6E,F), which agrees with ThT fluorescence assay results. We then quantitated the soluble hCT concentrations in the supernatant after disaggregation by using bicinchoninic acid (BCA) assay. After 48 h of pre-incubation, the concentration of soluble hCT in the supernatant was lower than 20 μg/mL (Fig. 6G), whereas the equimolar amount of EGCG increased hCT concentration to *ca.* 50 μg/mL (*P* < 0.05; Fig. 6H,I). Equimolar amounts of Mag and Hon showed no obvious disaggregation effect, whereas at higher concentrations (3:1 and 5:1) significantly increased concentration of soluble hCT in the supernatant (more than 100 μg/mL) were observed after 48 h of incubation with two compounds (Fig. 6H,I).

### Magnolol and honokiol both directly bound to hCT

To further determine the intermolecular interactions between hCT and compounds, isothermal titration calorimetry (ITC) was used to measure the binding affinities of Mag and Hon to hCT as described^59^. We found that Mag bound to hCT with a K_b_ of (3.81 ± 1.28) × 10^5^ M^−1^, while Δ*H* and Δ*s* were −831.3 ± 107.7 cal/mol and 22.7 cal/mol/deg, respectively (Figure S3A). Hon bound to hCT with a K_b_ of (1.54 ± 0.395) × 10^5^ M^−1^, while Δ*H* and Δ*s* were −907.5 ± 73.22 cal/mol and 20.7 cal/mol/deg, respectively (Figure S3B). ITC measurements also indicated Mag bound to hCT at a 1:1 stoichiometry, while Hon interacted with hCT at a 3:1 stoichiometry.

## Discussion

The toxic aggregation of amyloidogenic proteins into toxic conformers, not only causes diseases but also limits the clinical application of many protein-based drugs^60^. A number of compounds have thus been screened or designed as amyloid inhibitors^17^,^61^, among which polyphenols have been extensively reported^17^,^62^. Here, Mag and Hon, two biphenyl backbone containing polyphenol compounds, were found to effectively inhibit the aggregation of hCT. Mag and Hon both directly bound to hCT (Figure S3), dose-dependently suppressed hCT aggregation (Fig. 2), and could disassemble preformed hCT aggregates (Fig. 6).

Oligomeric intermediates formed during the aggregation have been considered as the most toxic conformers^10^,^11^. Therefore, a number of inhibitors have been designed to disrupt the oligomerization step^63^,^64^. In our study, Mag and Hon were found to effectively inhibit both the oligomerization and fibrillation of hCT (Fig. 7). The presence of 3- and 5-fold of Mag and Hon prolonged the aggregation lag time of hCT, implying they may affect aggregation at the initial oligomerization stage (Fig. 2A,B). Further immuno-dot blot and DLS assays demonstrated that both compounds inhibited the formation of hCT oligomers and fibrils (Fig. 3,4), and reduced the membrane disruption and cytotoxicity induced by hCT oligomers and fibrils (Fig. 5).

**Figure 1:**
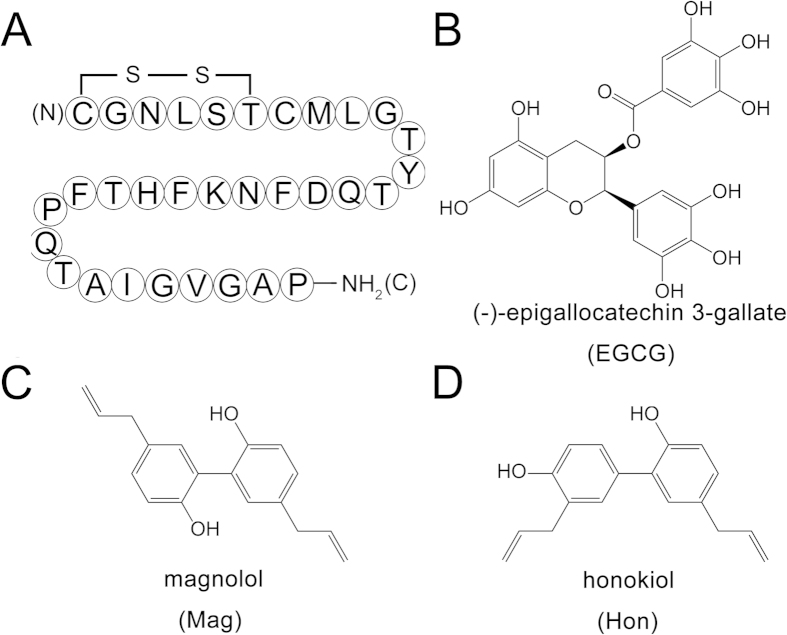
Structures of hCT and compounds. (**A**) Primary sequence of hCT with a disulfide bridge between Cys-1 and Cys-7 and C terminus amidated; (**B–D**) Chemical structures of EGCG (**B**), magnolol (**C**) and honokiol (**D**).

As generally acknowledged, proteins aggregate mostly through aromatic and hydrophobic interactions^65^,^66^, and aromatic interactions also play important role in the fibril formation of human calcitonin^67^; disrupting such interactions may thus suppress amyloid aggregation. NMR investigation had demonstrated EGCG bound to hCT through intermolecular π-π stacking, therefore inhibiting the aggregation of hCT^55^. By using ITC assay, we found Mag and Hon both bound to hCT; considering the polyphenol structure of these two molecules (Fig. 1C,D), we speculated that Mag and Hon may also bind to hCT through aromatic interaction, and suppressed amyloid formation of hCT.

**Figure 2:**
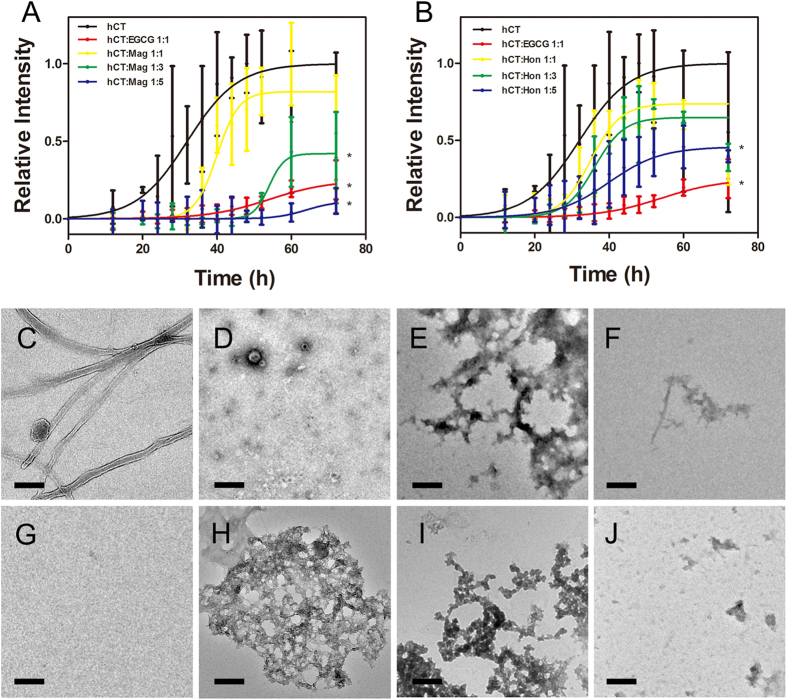
Inhibitory effects of compounds on hCT amyloid formation. (**A-B**) ThT fluorescence of hCT aggregation in the absence or presence of different ratios of magnolol (**A**) and honokiol (**B**). *P < 0.05; (**C–J**) TEM images of hCT aggregates in the absence or presence of different compounds. (**C**) hCT alone; (**D**) 1:1 mixture of hCT and EGCG; (**E–G**) Mixtures of hCT and magnolol with molar ratios of 1:1 (**E**), 1:3 (**F**) and 1:5 (**G**); (**H–J**) Mixtures of hCT and honokiol with molar ratios of 1:1 (**H**), 1:3 (**I**) and 1:5 (**J**). Scale bar represents 200 nm.

**Figure 3:**
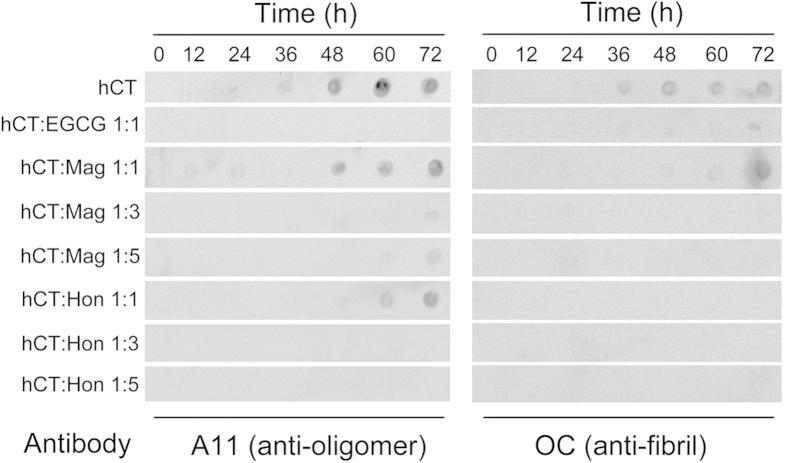
Inhibitory effects of magnolol and honokiol on hCT oligomerization and fibrillation detected by dot blot.

**Figure 4:**
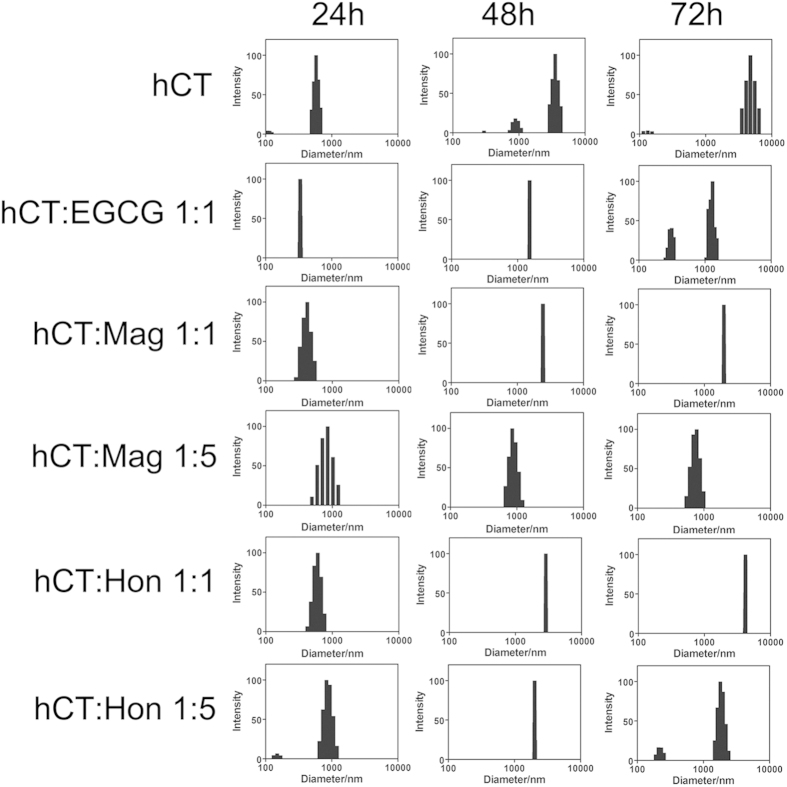
Size distribution of hCT aggregates detected by dynamic light scattering in the absence or presence of different compounds.

**Figure 5:**
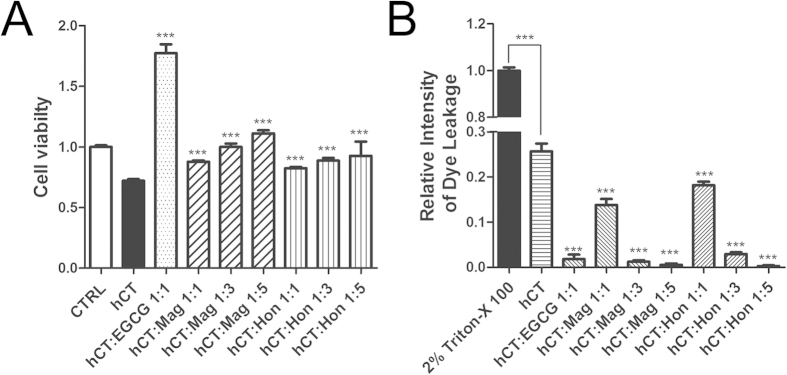
Effects of magnolol and honokiol on cytotoxicity and membrane disruption caused by hCT aggregates. (**A**) Cell viability of SH-SY5Y cells in the absence or presence of different compounds determined by MTT-based assay; (**B**) Dye leakage levels induced by hCT in the presence of compounds. 0.2% Triton X-100 was used as the positive control. ***P < 0.05.

**Figure 6:**
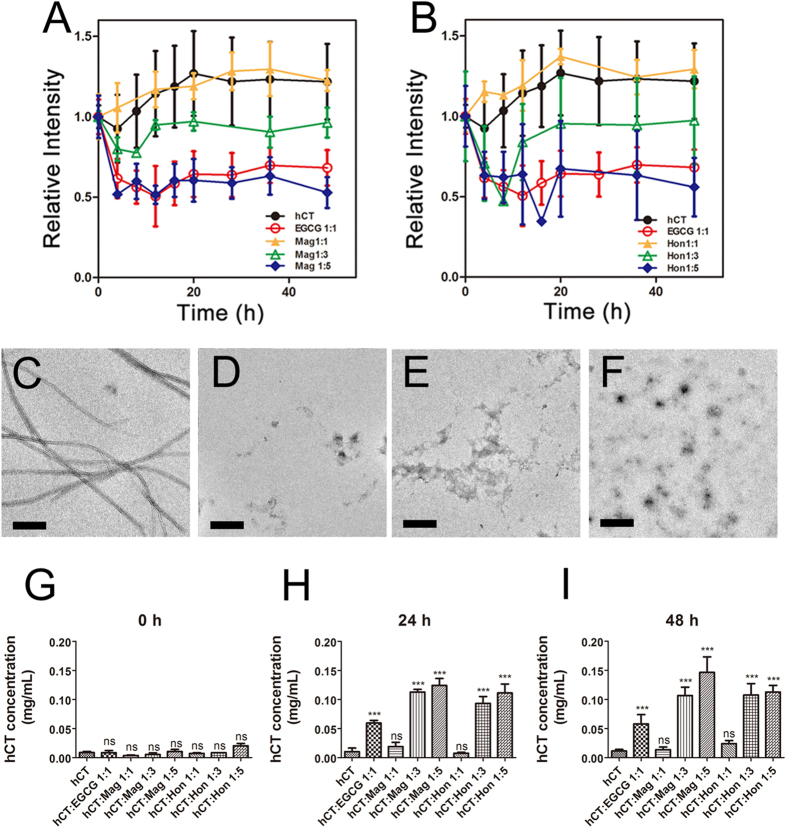
Disaggregation effects of magnolol and honokiol on hCT aggregates. (**A,B**) ThT fluorescence of disaggregation effects of magnolol (**A**) and honokiol (**B**). (**C–F**) TEM images of disaggregation effects of different compounds. (**C**) hCT incubated for 96 h; (**D**) hCT aggregates incubated with equimolar of EGCG for 48 h; (**E–F**) hCT aggregates incubated with 5-fold amounts of magnolol (**E**) and honokiol (**F**) for 48 h; Scale bar represents 500 nm. (**G–I**) Quantitative analysis of hCT concentration in the supernatant after disaggregation for 0 h (**G**), 24 h (**H**) and 48 h (**I**) in the presence of different compounds determined by bicinchoninic acid (BCA) assay.

**Figure 7:**
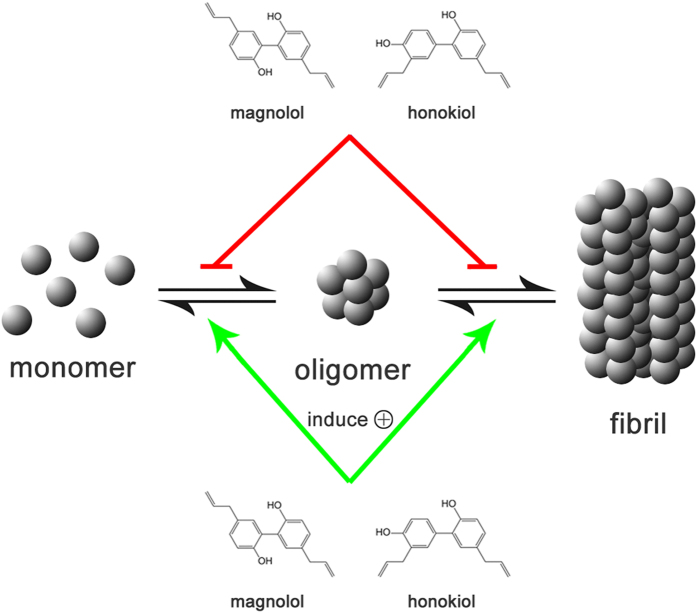
A schematic representation of how magnolol and honokiol affected hCT aggregation.

Compared with Hon, Mag displayed a similar inhibitory effect at a low equimolar concentration (1:1), but stronger inhibitory effects at high concentrations (3:1 and 5:1). ITC results also indicated Mag bound to hCT with a higher binding constant compared to Hon (Figure S3). This may due to the slight structural difference between two compounds. With two adjacent hydroxyls, Mag is more likely to form intramolecular hydrogen bonds, which causes a smaller angle between two benzene rings compared to Hon^68^,^69^. When binding to hCT molecules, a larger angle between two benzene rings may produce greater steric hindrance, which may explain the relatively lower potency of Hon compared to Mag on inhibiting hCT aggregation. It is also noted that Mag and Hon may inhibit hCT aggregation through different mechanisms. ThT fluorescence assay suggested that Mag increased lag time, and inhibited aggregation from oligomerization stage, whereas Hon showed little effects on increasing lag time although it decreased the fibril elongation rate (Fig. 2A,B). ITC assay also suggested Mag and Hon bound to hCT at a 1:1 and 3:1 stoichiometry, respectively (Figure S3), possibly indicating different binding sites on hCT between two compounds. Notably, in the dot blot assay, honokiol showed a stronger inhibitory effect compared with magnolol, especially at an equimolar ratio; this may also due to different interactions between two compounds, which leads to the different conformations formed during aggregation, and interferes the dot blot results. However, the molecular mechanism of inhibitory effects of Mag and Hon on hCT amyloid formation remained to be clarified, and it will be of great interest to detect monomer and polymers formed during aggregation with specific monoclonal antibody by using western blot approach as Tufail, S. *et al.* described^70^. It will also be useful to probe the exact membrane protection mechanisms of Hon and Mag by using biophysical techniques, particularly, with the use of high-resolution solids-state NMR approaches^50^,^55^,^71^,^72^,^73^.

In summary, our studies indicated Mag and Hon inhibited the oligomerization and fibrillation of hCT, disassembled hCT aggregates at higher molar ratios, and more importantly, reduced membrane disruption and cytotoxicity induced by hCT aggregates. Our results suggested a potential anti-amyloidogenic property of Mag and Hon, and polyphenols with a biphenyl backbone may further be explored to design effective anti-amyloidosis compounds.

## Methods

### Materials

Human calcitonin (>95%) was obtained from GL Chemicals Ltd. (Shanghai, China). Mag, Hon and EGCG were from Aladdin-reagent (Shanghai, China). Anti-oligomer antibody (OC), anti-fibril antibody (A11) and anti-rabbit IgG were obtained from Merck Millipore (Billerica, USA). Thioflavin-T (ThT), 2-Oleoyl-1-palmitoyl-snglycero-3-phospho-rac-(1-glycerol) sodium salt (POPG) and carboxyfluorescein were from Sigma-Aldrich (St. Louis, USA). SH-SY5Y cells were obtained from the China Center for Type Culture Collection (CCTCC). All other chemical reagents were of the highest grade available.

### Sample preparation

hCT aggregation was conducted with the protein misfolding cyclic amplification (PMCA) approach as previously described^74^,^75^. Briefly, hCT was dissolved in 50 mM PBS buffer (pH 7.4) containing 100 mM NaCl to a final concentration of 25 μM. Peptide solutions were incubated in eppendorf tubes containing 37.9 ± 0.7 mg of 1.0 mm Zirconia/Silica beads (Gong Tao INC., Shanghai, China). Mag and Hon were freshly prepared and added at different final concentrations. Samples were incubated at 37 °C and sonicated with a SB25-12DTD sonicator (Scientz Biotechnology, China) with an output power of 90% for 30 s every 4 h. For disaggregation assay, hCT was pre-incubated for 48 h to reach the plateau stage, followed by adding compounds at different ratios, and then further incubated for another 48 h.

### Thioflavin-T (ThT) fluorescence assay

ThT assay was performed on a Hitachi FL-2700 fluorometer. The excitation and emission wavelength were set at 450 nm and 482 nm, respectively, and the curves were fitted as we previously described^76^. All experiments were repeated for at least three times.

### Dot blot assay

Dot blot assay was performed as we previously described^77^. Briefly, samples were spotted on nitrocellulose membranes (Biorad, Hercules, USA) and dried for 30 min. The blots were blocked in 10 % nonfat milk TBST for 30 min at room temperature, and then incubated with A11 and OC antibodies for 4 h at 4 °C, followed by co-incubating with secondary anti-rabbit IgG antibody for 2 h at room temperature. The blots were further incubated with ECL reagent (Millipore, Billerica, USA) for 2 min, and developed.

### Transmission electronic microscopy (TEM) assay

TEM assay was performed as we previously described^76^. 5 μL of each sample was dropped onto a 300-mesh Formvar-carbon-coated copper grid and air dried. Samples were then stained with 1% uranyl formate. Images were observed under a FEI Tecnai G2 20 U-TWIN transmission electronic microscope (Hillsboro, OR, USA).

### Dynamic light scattering (DLS) analysis

Dynamic light scattering was performed on a zeta pals potential analyzer (Brookhaven Instruments, USA) as we described before^78^. Briefly, samples were measured with a scattering angle of 90^o^. Each sample was scanned for three times (1 min/scan), and the data were analyzed by the multimodal size distribution (MSD).

### Dye leakage assay

Dye leakage assay was performed as we previously described^35^. POPG was dissolved in chloroform and the solvent was evaporated to form lipid films. Carboxyfluorescein was dissolved in 50 mM PBS to a final concentration of 40 mM, and was then added into the lipid films to form vesicles. Vesicles containing carboxyfluorescein were further purified by a PD-10 column (Sangon Biotech., Shanghai, China). Samples pre-incubated for 48 h were then added into the POPG vesicles at a final peptide concentration of 1 μM, and fluorescence intensities were measured after mixing for 100 s with excitation and emission wavelengths set at 493 and 518 nm, respectively. The vesicles were then treated with 0.2% Triton X-100, which completely disrupted the vesicles. All experiments were repeated for at least three times.

### MTT cell toxicity assay

MTT-based cell toxicity assays were performed as we previously described^79^. SH-SY5Y cells were cultured in DMEM high glucose medium containing 10% FBS, 1% penicillin-streptomycin solution and 1% sodium pyruvate. Cells were plated in 96-well plates at a density of 5 × 10^3^ cells/well and cultured for 24 h. After addition of fresh medium containing hCT samples which were pre-incubated for 48 h with or without compounds, cells were further incubated for another 24 h. 10 μL of MTT (5 mg/mL) were then added into the wells, followed by the addition of formazan buffer, and the absorbance was measured at 570 nm.

### Bicinchoninic acid (BCA) assay

80 μL disaggregation samples were centrifuged at 12,000 rpm for 10 min, and 10 μL of supernatants were withdrawn and added into 96-well plates. 100 μL of BCA reagent A and B (Beyotime, China) mixtures were then added, and samples were incubated at 37 °C for 30 min. The absorbance was measured at 562 nm, and the concentration of hCT was calculated by referring to standard curve. All experiments were repeated for at least 3 times.

### ITC measurements

ITC measurements were performed on a Microcal VP-ITC titration calorimeter (GE Healthcare, USA) at 25 °C. hCT and compounds were dissolved in 50 mM PBS (pH 7.4, containing 100 mM NaCl), and solutions were degassed for 5 min by using ThermoVac (GE Healthcare, USA). hCT was used as a titrant in the sample cell at a concentration of 10 μM, while Mag (0.2 mM) and Hon (0.4 mM) were loaded into the syringe. Compounds were injected into hCT solution with 25 consecutive injections with stirring at 307 rpm. The first injection volume was 3 μL with 6-s duration, while the rest injection volume was 10 μL with injection duration of 20 s. Control experiments were carried out by injecting compounds into PBS, and results were analyzed with MicroCal ORIGIN software with curves fitted with a one set of sites model.

### Statistical analysis

Each experiment was repeated three times and the data were expressed as the mean ± SD. The Kruskal-Wallis test and the Mann-Whitney test were used to evaluate statistical significance. Difference was considered statistically significant at *P* < 0.05.

## Additional Information

**How to cite this article**: Guo, C. *et al.* Inhibitory effects of magnolol and honokiol on human calcitonin aggregation. *Sci. Rep.* **5**, 13556; doi: 10.1038/srep13556 (2015).

## Supplementary Material

Supplementary Information
