# Supplementary material for: Inhibitory effects of magnolol and honokiol on human calcitonin aggregation
Source: Sci Rep. 2015 Sep 1;5:13556. doi: 10.1038/srep13556 (PMC4555095; doi:10.1038/srep13556)
Supplement: Supplementary Information [file srep13556-s1.doc]

**Supplementary Information**

*for*

**Inhibitory effects of** **magnolol and honokiol on human calcitonin aggregation**

Caiao Guo^1^, Liang Ma^1^, Yudan Zhao^1^, Anlin Peng^2^, Biao Cheng^3^, Qiaoqiao Zhou^1^,

Ling Zheng^4^ & Kun Huang^1, 5*^

^1^ Tongji School of Pharmacy, Huazhong University of Science and Technology, Wuhan, Hubei, P. R. China, 430030;

^2^ Dept. of Pharmacy, The third hospital of Wuhan, Wuhan, Hubei, P. R. China, 430060;

^3^ Dept. of Pharmacy, Central Hospital of Wuhan, Wuhan, Hubei, P. R. China, 430014;

^4^ College of Life Sciences, Wuhan University, Wuhan, Hubei, P. R. China, 430072;

^5^ Centre for Biomedicine Research, Wuhan Institute of Biotechnology, Wuhan, Hubei, P. R. China, 430075;


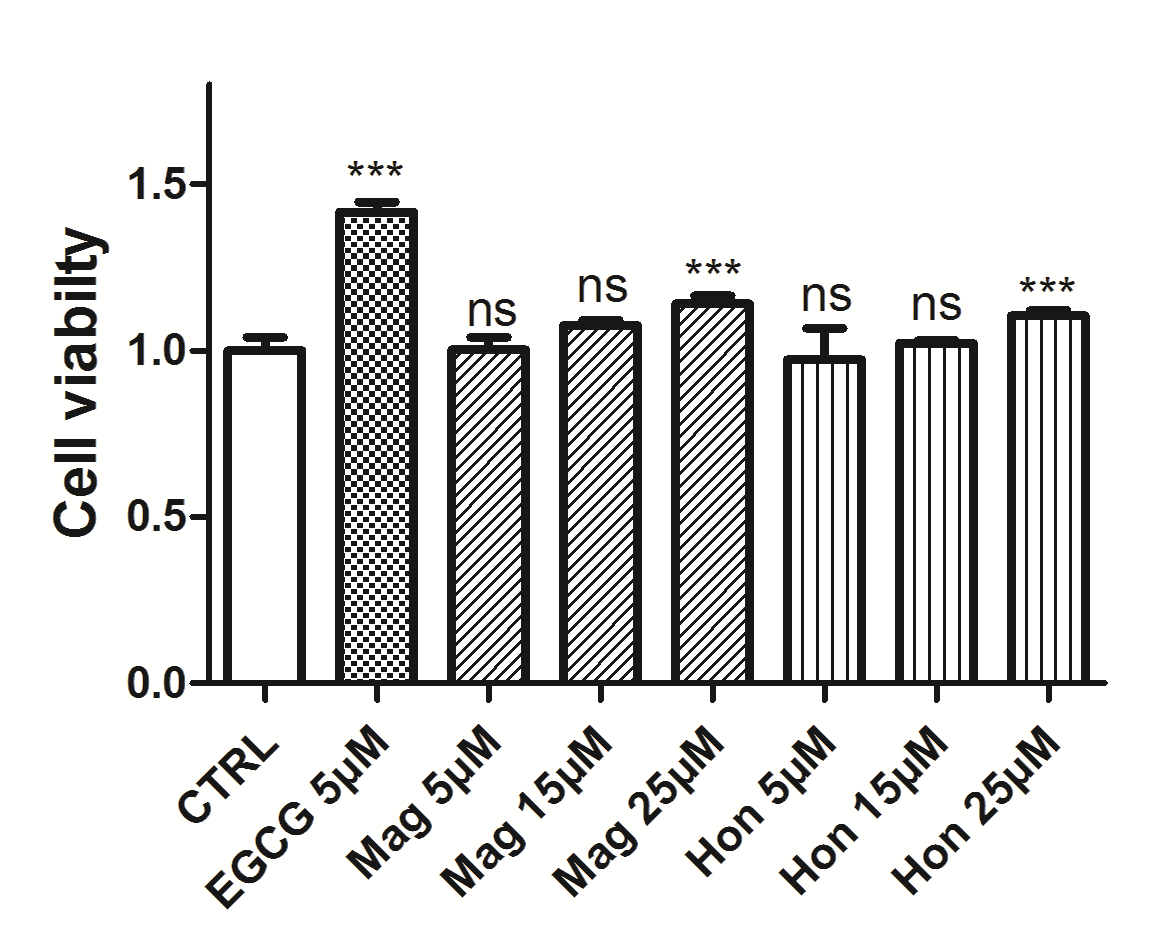


***Figure S1***

***Figure S1.*** The effects of compounds on SH-SY5Y cells viability determined by MTT. *** *P* < 0.05.


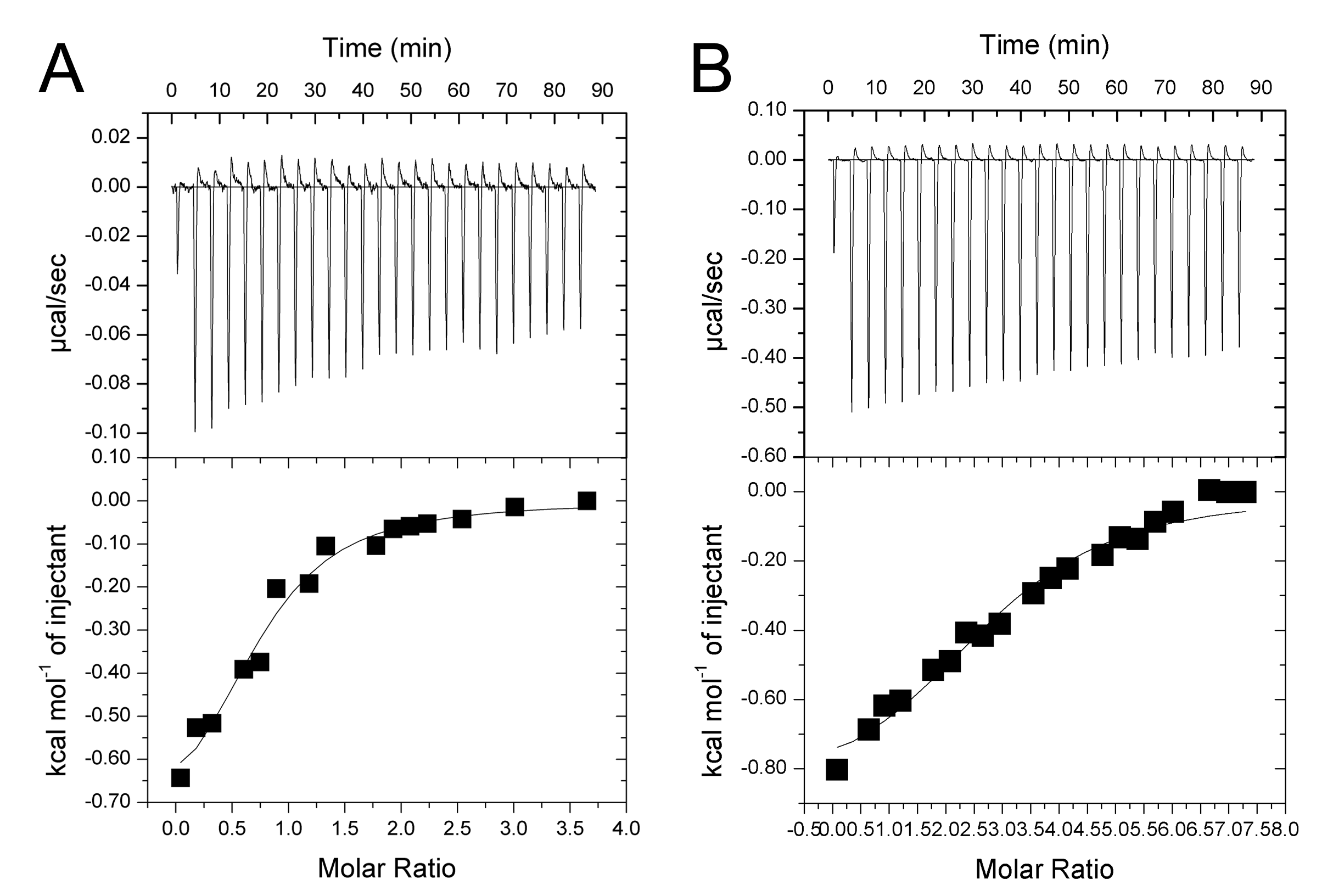


***Figure S2***

***Figure S2.*** ITC profiles for the binding of magnolol and honokiol to hCT at 25℃. (A) ITC data of magnolol (0.2 mM) injecting into hCT (10 μM); (B) ITC data of honokiol (0.4 mM) injecting into hCT (10 μM).
